# In-Situ Reaction Method to Synthetize Constant Solid-State Composites as Phase Change Materials for Thermal Energy Storage

**DOI:** 10.3390/ma14206032

**Published:** 2021-10-13

**Authors:** Bo Yang, Yang Liu, Wenjie Ye, Qiyang Wang, Xiao Yang, Dongmei Yang

**Affiliations:** Joint Laboratory of Regional Energy Internet Technology and Application of State Grid Corporation, State Grid Electric Power Research Institute, Nanjing 211106, China; liuyang9@sgepri.sgcc.com.cn (Y.L.); yewenjie@sgepri.sgcc.com.cn (W.Y.); wangqiyang@sgepri.sgcc.com.cn (Q.W.); yangxiao1@sgepri.sgcc.com.cn (X.Y.); yangdongmei@sgepri.sgcc.com.cn (D.Y.)

**Keywords:** thermal energy storage, in-situ reaction, 15CPCMs, thermophysical properties

## Abstract

The encapsulation and heat conduction of molten salt are very important for its application in heat storage systems. The general practice is to solidify molten salt with ceramic substrate and enhance heat conduction with carbon materials, but the cycle stability is not ideal. For this reason, it is of practical significance to study heat storage materials with a carbon-free thermal conductive adsorption framework. In this paper, the in-situ reaction method was employed to synthetize the constant solid-state composites for high-temperature thermal energy storage. AlN is hydrolyzed and calcined to form h-Al_2_O_3_ with a mesoporous structure to prevent the leakage of molten eutectic salt at high temperature. Its excellent thermal conductivity simultaneously improves the thermal conductivity of the composites. It is found that 15CPCMs prepared with 15% water addition have the best thermal conductivity (4.928 W/m·K) and mechanical strength (30.2 MPa). The enthalpy and the thermal storage density of 15CPCMs are 201.4 J/g and 1113.6 J/g, respectively. Due to the excellent leak-proof ability and lack of carbon materials, the 15CPCMs can maintain almost no mass loss after 50 cycles. These results indicate that 15CPCMs have promising prospects in thermal storage applications.

## 1. Introduction

With the rise of integrated energy in recent years, thermal energy storage systems have played an increasingly vital role in energy coupling to ensure the safe, economic and efficient supply of energy [1,2]. As a key component, thermal storage materials have received a great deal of attention from researchers [3,4,5]. Obviously, the most valuable and promising materials are based on latent thermal storage using phase change materials (PCMs) [6,7,8], which have a high heat storage density and constant temperature during the charge/discharge process [9,10]. Compared with hydrated salt and their mixture, molten salt is more attractive with the advantages of low subcooling, high melting temperature and high phase stability, which offer more potential for applications in solar thermal uses, waste heat recovery, steam production and so on [11,12].

However, limited by its low thermal conductivity, insurmountable thermal expansion and corrosion, there are still many difficulties for molten salt in practical use as a heat storage material [13,14]. To date, many efforts have been made to solve these problems, which can be summarized as follows: forming a skeleton by the use of chemical and physical compatible ceramic materials and enhancing the heat conductivity by the addition of thermal conductivity materials [15,16,17,18]. Awad [19] prepared nanosalts by dispersing nanoparticles in nitrate, whose total heat storage increased by 6% due to the formation of an interface layer. Porous Si_3_N_4_ ceramic acted as a heat-conducting skeleton material to prepare shape-stabilized phase change material in Wang’s work [15], and the finished product showed good thermal conductivity and cyclic stability. By inserting the mixture of salt into the cavities of diatomite, Miliozzi [20] fabricated solar salt/diatomite composite PCMs, which showed increased thermal and mechanical properties without any phase change material leakage. A shape-stable Na_2_CO_3_-K_2_CO_3_/CFA/EG composite phase change material was developed by Wang [21]. It was found that the addition of expanded graphite has little effect on the heat storage density but can greatly improve the thermal conductivity (3.182 W/m·K) of the composites. Sang [22] studied composite PCMs based on MgO and ternary carbonate, and the results showed that when the mass ratio of MgO and ternary carbonate was 1:1, the composite PCMs had the best energy storage performance and thermophysical properties. Ye [23] prepared CPCMs with MgO and multi-layer carbon nanotubes as a ceramic framework and thermal agent, respectively. The study found that the overall structure of the material is stable, and the thermal conductivity is significantly enhanced with increased amounts of multi-layer carbon nanotubes. The wettability of the molten salt on the ceramic and carbon materials was also studied [24], showing that the amount of carbon materials significantly affected the microstructure and properties of the composites. Li [25] employed a milling and sintering approach to fabricate MgO-based PCMs with different particle sizes and densities of MgO. They found that light and smaller MgO particles could provide better thermophysical and mechanical properties for PCMs and that graphite loading is the important factor affecting the thermal conductivity.

Though the research work in the above literature has solved the problems of the shaping and heat conduction of PCMs to a certain extent, there are still some defects left: the pure ceramic (<36 W/m·K) framework has good molten salt fixation ability but poor thermal conductivity [23], while carbon materials have good thermal conductivity but poor high temperature stability and molten salt dispersion [26]. Herein, an in-situ reaction method is proposed to synthetize constant solid-state CPCMs. AlN powder (up to 180 W/m·K) is used and partially hydrolyzed to form a ceramic framework, while the remaining powder acts as a thermal conductive material. In this way, a uniform and continuous thermal conduction skeleton of a ceramic matrix is formed, which means that the CPCMs can obtain higher thermal conductivity and cycle stability.

## 2. Materials and Methods

### 2.1. Raw Materials and Sample Preparation

**Scheme 1:**
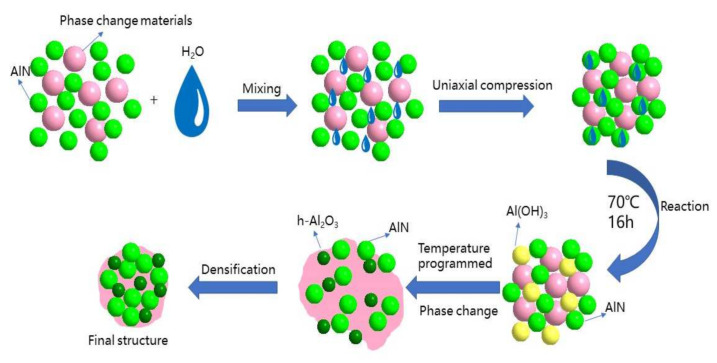
The synthesis process of the constant solid-state CPCMs.

Constant solid-state composites were obtained via an in-situ reduction method, as described in Scheme 1. The PCMs used in this study were carbonate-based eutectic salt made from a mixture of Li_2_CO_3_ (AR, Aladdin reagent Co., Ltd., Shanghai, China) and Na_2_CO_3_ (AR, Aladdin reagent Co., Ltd., Shanghai, China) with a mole ratio of 1:1. Ceramic powder (AlN, purity > 98%, Liaoning Desheng special ceramics manufacturing Co., Ltd., Tieling, China), with a particle size of about 10 μm, was chosen to produce the thermal conductive reinforced framework. All these raw materials were used without further purification.

To synthesize the composites, a certain amount of eutectic salt and AlN powder was mixed by the method of high-speed shear. Then, deionized water was added into the mixture homogeneously to prepare composite powder, which was shaped under a uniaxial press of 30 MPa for 3 min. The precursor was sealed and heated for 16 h at a temperature of 70 °C to ensure full reaction. In this way, green bulks were obtained. Finally, the green bulks were sintered by the following heating procedure: they were heated to 105 °C at 5 °C/min and held for 1 h; raised to 300 °C at 10 °C/min and held for 2.5 h; raised to 550 °C at 5 °C/min and held for 2 h; and finally cooled down to room temperature. The contrast samples were prepared by using Al_2_O_3_ nano powder and flake graphite as skeleton and thermal conductivity materials, respectively.

### 2.2. Material Characterization

The phase structures of materials were characterized by X-ray diffraction (XRD) (D8 advance, Bruker, Karlsruhe, Germany) with Cu Kα radiation (λ = 1.5418 Å, 40 kV, 40 mA). The morphologies were observed by scanning electron microscopy (SEM) (Nova Nano 450, FEI, Hillsboro, OR, USA) with energy dispersive X-ray analysis (EDS, Octane Super, EDAX, Pleasanton, CA, USA). The chemical state of materials was investigated by X-ray photoelectron spectroscopy (XPS) (K-Alpha 1063, Thermo Scientific, Waltham, MA, USA). The specific surface area and pore volume of the materials were analyzed with the BET method (ASAP 2460, Micromeritics, Norcross, GA, USA). The measurements of melting temperature, latent heat and thermal stability were performed with a simultaneous thermal analyzer (TG-DSC) (STA449F3, Netzsch, Waldkraiburg, Germany) from room temperature to 600 °C at a heating rate of 10 °C/min under air. The specific heat capacity of samples was tested by the sapphire method on the same instrument. The thermal conductivity of the samples was detected by a thermal constant analyzer (TPS 2500S, Hot Disk, Uppsala, Sweden) at room temperature. The mechanical strength of the CPCMs was measured by the electronic universal material testing machine (CMT4202, Shenzhen Xinsansi Material Test Co. Ltd., Shenzhen, China).

### 2.3. Thermal Cycling Test

To obtain the thermal cycling performance of the CPCMs, all of the samples were loaded into corundum crucibles and then placed into the muffle furnace. In the test, the temperature was raised to 600 °C at a heating rate of 10 °C/min for 1 h to ensure the completion of the phase change process and then downed with the furnace. This was recorded as a cycle. A total of 50 cycles were carried out and the mass data were recorded every 5 times.

## 3. Results and Discussion

### 3.1. Mechanical Strength and Thermal Conductivity of CPCMs

**Figure 1:**
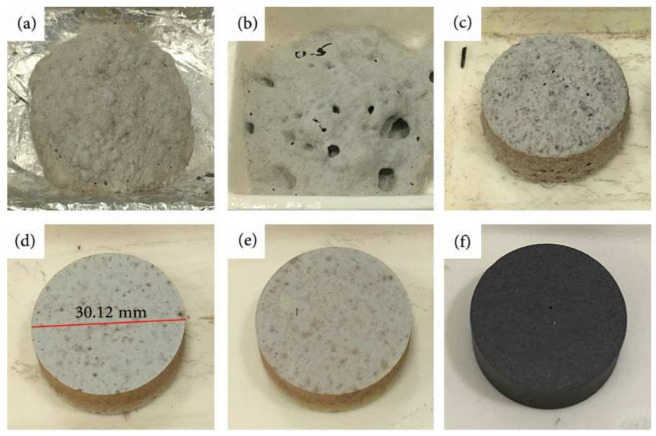
Photographs of the CPCMs made with different water addition: (**a**) 0%, (**b**) 5%, (**c**) 10%, (**d**) 15%, and (**e**) 20% water and (**f**) the contrast sample.

Figure 1 shows the appearances of the CPCMs made with different levels of water addition and the contrast sample. As shown in Figure 1a,b, there was a serious leakage for 0CPCMs and 5CPCMs due to the inability to produce enough skeleton materials. Though 10% water addition can allow the 10CPCMs to be shaped, the escape of phase change materials at high temperature cannot be prevented. For CPCMs made with more than 15% water addition, they have perfect appearances without any leakage. As above, this indicates that with at least 15% water addition, the produced CPCMs can wrap the eutectic salt. The contrast sample exhibits an excellent appearance, similar to that of 15CPCMs and 20CPCMs, which shows the good adsorption capacity of nano Al_2_O_3_.

The physical parameters of density, mechanical strength and thermal conductivity are of great importance to CPCMs for practical use. Thus, the stable samples were chosen for further study, as shown in Table 1. Comparing 15CPCMs with 20CPCMs, it can be found that water addition has an obvious effect on the density, which can be attributed to the hydrolysis of AlN powder. As the control agent of reaction, the amount of water determines the rate of reaction and the amount of product and then affects the density of CPCMs. The density is closely related to mechanical strength and heat conduction, so the three technical indicators of 15CPCMs are higher than 20CPCMs. Due to the thermal inertia and fluffiness of nano Al_2_O_3_, although graphite has good thermal conductivity and compaction density, the contrast sample still displays worse performance than 15CPCMs.

### 3.2. Chemical Compatibility and Microstructures of CPCMs

**Figure 2:**
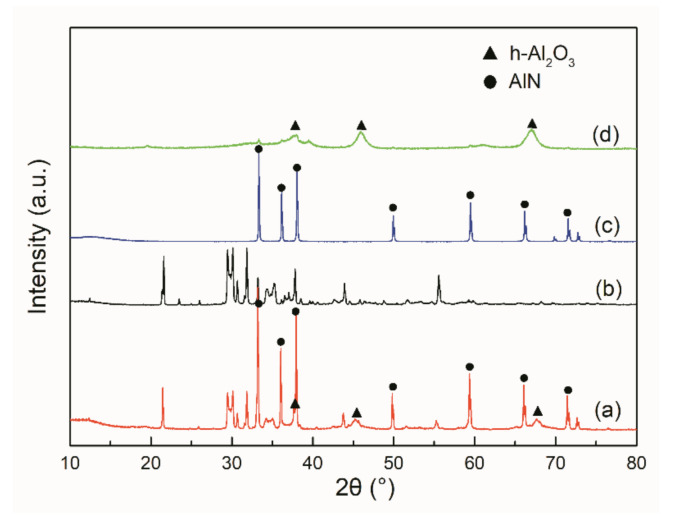
XRD patterns of (**a**) 15CPCMs, (**b**) eutectic salt, (**c**) AlN and (**d**) h-Al_2_O_3_.

Figure 2 shows the XRD patterns of 15CPCMs, eutectic salt, AlN and h-Al_2_O_3_, where h-Al_2_O_3_ represents the result of AlN after hydrolysis and calcination. The eutectic salt was made from Li_2_CO_3_-Na_2_CO_3_ according to the mole ratio of 1:1, which matches well with standard LiNaCO_3_ (JCPDS No. 34-1193). As shown in Figure 2a, the characteristic peaks of eutectic salt, AlN and h-Al_2_O_3_ are all observed in the XRD pattern of 15CPCMs, which proves that part of AlN reacts to generate h-Al_2_O_3_ during the preparation process. Besides, no new peaks could be detected, indicating that there were no chemical reactions among eutectic salt, AlN and h-Al_2_O_3_. It is worth mentioning that the crystallinity of h-Al_2_O_3_ is poor according to the broad band of the XRD pattern.

**Figure 3:**
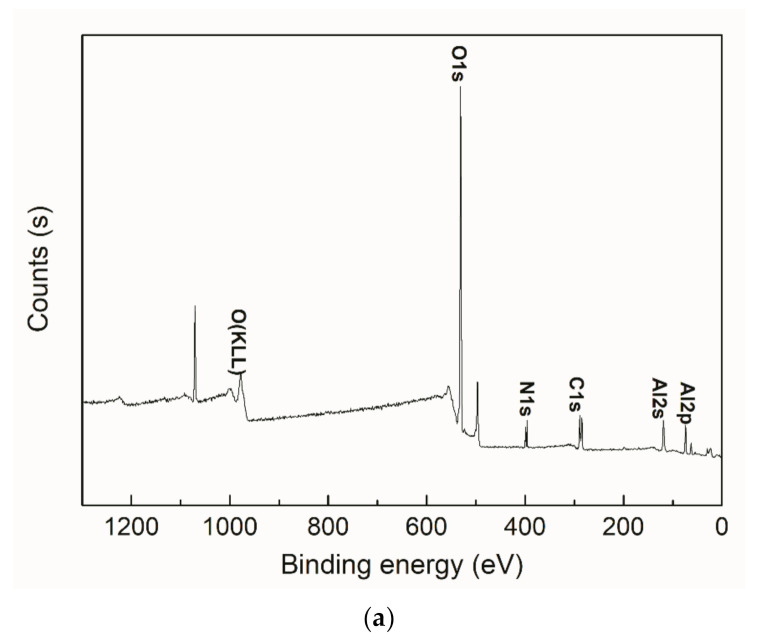

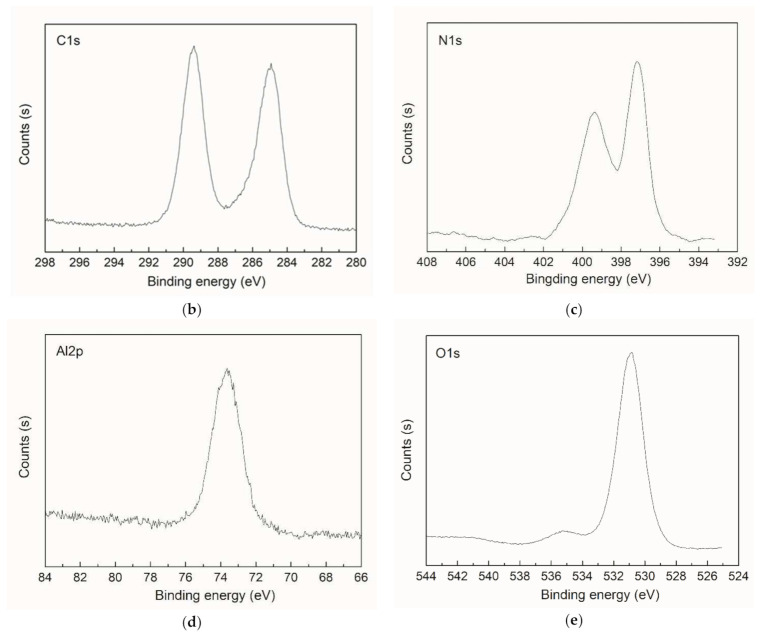
Wide-scan XPS spectrum of 15CPCMs (**a**). High-resolution scan for (**b**) C1s, (**c**) N1s, (**d**) Al2p and (**e**) O1s core levels.

The elemental compositions of the 15CPCMs were examined by XPS analysis. As shown in Figure 3a, there are five distinct peaks in the wide-scan XPS spectrum, illustrating the existence of C, N, Al and O atoms. The group peaks near 979.7 eV are the auger electron spectra of oxygen, which is the KLL series. The C1s region consists of two strong peaks at 285.0 eV and 289.4 eV, which can be attributed to sp2-bonded carbon and MCO_3_, indicating that the composites contain carbonate. The N1s core level spectrum at 397.1 eV and 399.4 eV is related to AlN and the binding energy of O1s at 531.1 eV indicates the presence of Al_2_O_3_. Furthermore, the binding energy peak at 73.8 eV represents the Al bonded to N, which is consistent with the detection of N1s. This testing result reveals that the components of 15CPCMs include AlN, Al_2_O_3_ and carbonate.

**Figure 4:**
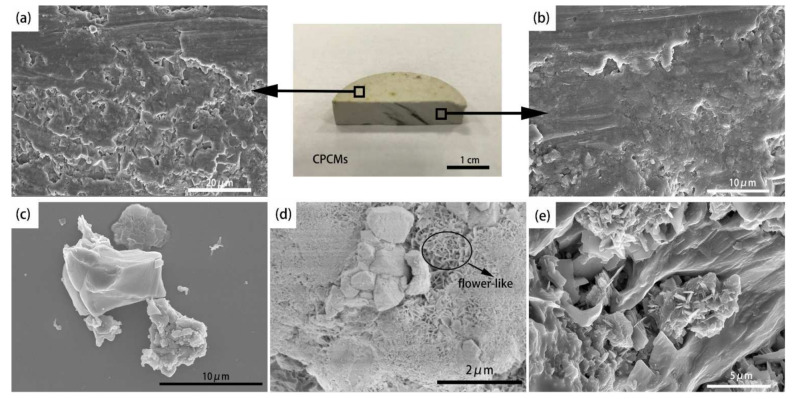
SEM images of 15CPCMs and components: (**a**) upper surface, (**b**,**e**) section of 15CPCMs, (**c**) AlN and (**d**) h-Al_2_O_3_.

Figure 4d gives the morphology of h-Al_2_O_3_, which presents a flower-like appearance with the petals size below 1 μm, and many pores are formed among the petals to accommodate the phase change materials. Thanks to this microstructure, the upper surface and section of 15CPCMs show a dense appearance without holes (Figure 4a,b), indicating that there is no eutectic salt volatilization at high temperature. From Figure 4e, it can be further observed that the eutectic salt is enwrapped by the h-Al_2_O_3_. Compared to this, AlN shows a smooth appearance with no pores (Figure 4c). Therefore, the capillary force provided by the molten salt is insufficient to hold the AlN together [24], and the 0CPCMs prepared with it cannot be formed after sintering.

**Figure 5:**
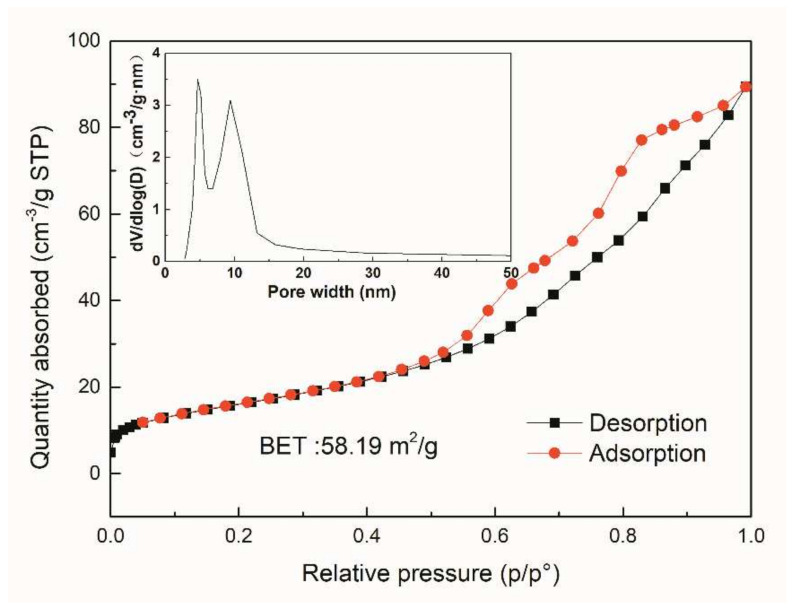
N_2_ adsorption–desorption isotherms and pore size distribution of h-Al_2_O_3_.

The N_2_ adsorption and desorption were carried out for h-Al_2_O_3_ to evaluate the permanent porosity. The h-Al_2_O_3_ exhibits the reversible type IV isotherm, which is one of the main characteristics of mesoporous materials (Figure 5). The surface area of h-Al_2_O_3_ was calculated to be 58 m^2^g^−1^ using the Brunauer–Emmett–Teller (BET) model. The pore size distribution of h-Al_2_O_3_ can also be observed in Figure 5, showing peak pore sizes of 5 nm and 13 nm. Obviously, this is within the size range of mesopores, indicating that the material has a mesoporous structure. This is the reason why the 15CPCMs made with h-Al_2_O_3_ exhibit excellent performance.

### 3.3. Thermophysical Properties of the Composites

**Figure 6:**
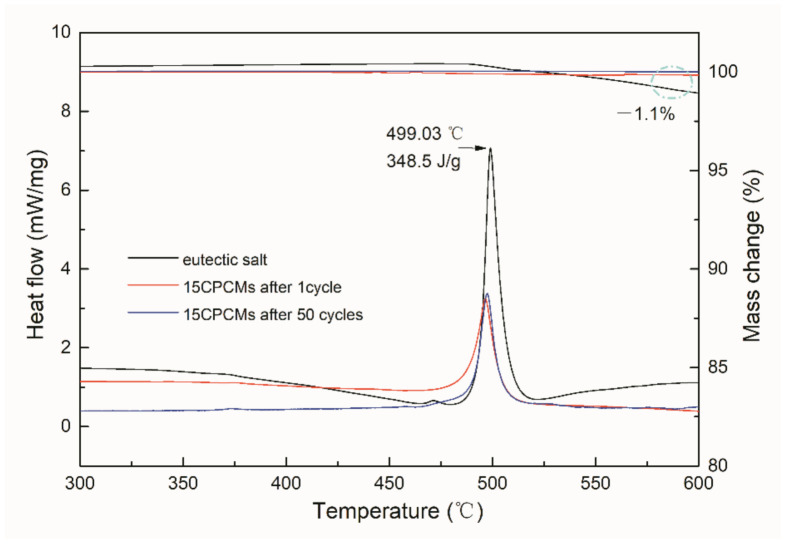
TG-DSC curves for eutectic salt and 15CPCMs after 1 cycle and 50 cycles.

The TG–DSC curves for the eutectic salt and 15CPCMs after 1 cycle and 50 cycles are given in Figure 6. It can be seen from the TG curves that there is an obvious mass loss for eutectic salt at 600 °C, indicating that although the decomposition temperature of a single component is not reached, the eutectic salt has begun to volatilize at this temperature, while the 15CPCMs have almost no mass loss at 600 °C, which proves that the formation of capillary force and surface tension by the mesoporous structure is of positive significance for the immobilization of liquid eutectic salt. From the DSC curves, the enthalpy of eutectic salt is 348.5 J/g, while the enthalpy of 15CPCMs is 201.4 J/g, which is about 57.8% of the former. The encapsulation amount of eutectic salt is higher than that reported in the literature [22,24]. Even after 50 thermal cycles, the enthalpy of 15CPCMs is hardly changed, which illustrates their good thermal stability. The melting temperature peak of 15CPCMs is ∼2.4 °C lower than that of eutectic salt, which may be associated with the surface energy differences. This is consistent with the results of Li’s research [25].

**Figure 7:**
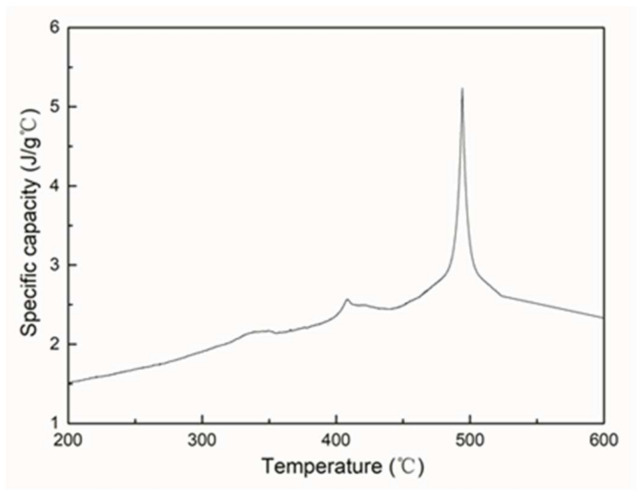
Specific heat capacity curve of 15CPCMs.

Thermal storage density is an important parameter to measure the practicability of thermal storage materials. To calculate the thermal storage density, it is necessary to consider both the latent heat of the eutectic salt and the sensible heat of the composite material. The calculation formula for thermal storage density is shown in Equation (1) in the temperature range from *T*_0_ to *T*_1_:

(1) $$Q=\Delta H_{lat}+\int_{T_{0}}^{T_{1}}C_{p}d_{t}\;$$

where *Q* is the total thermal energy stored in the composites per unit mass. Δ*H_lat_* stands for the latent heat of molten salts and *C_p_* represents the specific heat capacity of the composites, which are all detected by the simultaneous thermal analyzer. Figure 7 gives the specific heat capacity curve of 15CPCMs from 200 °C to 600 °C. Therefore, within this service temperature range, the heat storage density value of 15CPCMs is as high as 1113.6 J/g. Compared to the ternary carbonates/MgO composites obtained by Sang [22], whose thermal storage density is 781.4 kJ/kg, the result is obviously more attractive.

**Figure 8:**
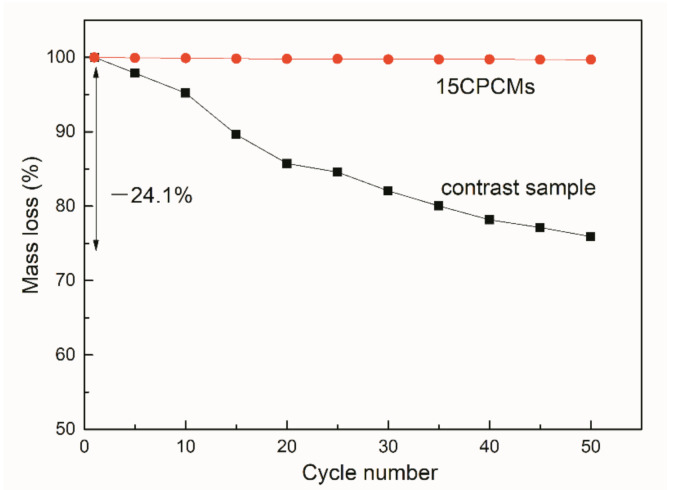
Weight loss curves of 15CPCMs and contrast sample.

**Figure 9:**
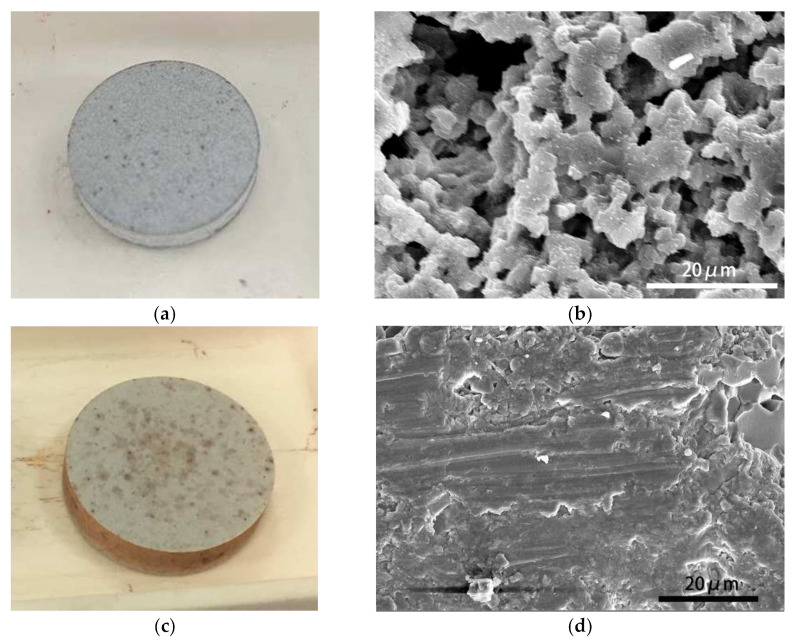
Morphology of contrast sample and 15CPCMs after 50 cycles: (**a**) photograph of contrast sample, (**b**) SEM image of contrast sample, (**c**) photograph of 15CPCMs, (**d**) SEM image of 15CPCMs.

Figure 8 gives the mass change of 15CPCMs and the contrast sample. The mass of 15CPCMs is almost unchanged after 50 cycles, indicating that the eutectic salt does not leak out from the ceramic matrix heat conduction skeleton, which can be proved by Figure 9c,d. This is consistent with the TG-DSC test results, and the enthalpy is roughly equal before and after cycling. However, the contrast sample loses about a quarter of its weight after 50 cycles. The reason for this can be attributed to the following: firstly, the continuous oxidation of graphite causes weight loss; secondly, the pores generated after graphite oxidation make the eutectic salt easy to volatilize, resulting in weight loss. These can be proved by Figure 9a,b. After 50 cycles, the contrast sample turns white due to the oxidation of graphite and presents an unsmooth and porous morphology.

## 4. Conclusions

Constant solid-state CPCMs with a carbon-free thermal conductive adsorption framework were prepared by an in-situ reaction method. In the CPCMs, carbonate-based eutectic salt (LiNaCO_3_) functions as the PCMs, while AlN serves as a thermal conducting material and generates h-Al_2_O_3_ through hydrolysis and calcination, which acts as a skeleton material for shape stabilization. We found that 15% of water addition can allow CPCMs to form and obtain the best performance, showing a mechanical strength of 30.2 MPa and a thermal conductivity of 4.928 W/m·K. Further study reveals that h-Al_2_O_3_ is a mesoporous structure that can produce enough capillary force and surface tension to prevent the leakage of molten eutectic salt at high temperature, which is the reason why the 15CPCMs exhibit excellent performance. The thermophysical test results show that the enthalpy and the thermal storage density of 15CPCMs are 201.4 J/g and 1113.6 J/g, respectively. After 50 cycles, the 15CPCMs have almost no mass loss and exhibit excellent thermal stability, which is much better than that of carbon-containing composites. All these factors prove that 15CPCMs are a promising high-temperature thermal energy storage material.

## Figures and Tables

**Table 1:** Density, mechanical strength and thermal conductivity of the composite materials.

Name	ρ (g/cm^3^)	P (MPa)	K (W/m·K)
15CPCMs	1.86	30.2	4.928
20CPCMs	1.73	26.3	4.149
Contrast sample	1.69	18.5	4.465

## Data Availability

Available upon request from the corresponding author.
